# Clinical characteristics and prognosis of amyotrophic lateral sclerosis with autoimmune diseases

**DOI:** 10.1371/journal.pone.0266529

**Published:** 2022-04-07

**Authors:** Jin-Yue Li, Xiao-Han Sun, Dong-chao Shen, Xun-Zhe Yang, Ming-Sheng Liu, Li-Ying Cui

**Affiliations:** 1 Department of Neurology, Peking Union Medical College Hospital, Chinese Academy of Medical Science & Peking Union Medical College, Beijing, China; 2 Neuroscience Center, Chinese Academy of Medical Sciences, Beijing, China; First Affiliated Hospital of Dalian Medical University, CHINA

## Abstract

**Introduction:**

The occurrence of autoimmune diseases (AIDs) in amyotrophic lateral sclerosis (ALS) patients is widely reported, but little is known about the associated clinical phenotype. This study aims to evaluate the clinical features and prognosis of ALS patients with AID.

**Methods:**

This retrospective study was based on the ALS Registry dataset of Peking Union Medical College Hospital from 2013 to 2020. Clinical features and inflammatory biomarkers at registration were compared between ALS patients with coexisting AIDs and those without (controls). The medical records of immunotherapy were also collected. The Kaplan–Meier method and Cox proportional hazard model were used to study the survival of ALS patients.

**Results:**

There are 26 (1.6%) ALS patients with AIDs in our database. The ALS patients with AIDs had older ages at onset and poorer respiratory function than controls (*p*<0.05). After propensity score matching by sex, onset age, and disease duration, the difference in respiratory function remained significant between groups. We found no differences in overall survival between ALS patients with and without AIDs before and after matching (*p* = 0.836; *p* = 0.395). Older age at onset, rapid disease progression, and lower erythrocyte sedimentation rate (ESR) were associated with shorter survival (*p*<0.05). Among ALS patients with AIDs, 8 (30.8%) had a history of immunotherapy and showed slightly prolonged survival compared with those without immunotherapy, but the results did not reach statistical significance (*p* = 0.355).

**Conclusions:**

Patients with coexisting ALS and AIDs had older onset age and poorer respiratory function but similar overall survival than those with pure ALS.

## Introduction

Amyotrophic lateral sclerosis (ALS) is a rare neurodegenerative disease characterized by the progressive loss of upper and/or lower motor neurons, leading to respiratory failure or death within 3 to 5 years after symptom onset. The majority of ALS patients have sporadic (SALS), while less than 10% of patients have a positive family history (FALS) with probable mutations in genes such as SOD1, C9orf72, TARDBP, and FUS [1]. Until now, the pathophysiology of ALS has not been well understood, but it has now been accepted that inflammation might play an important role in the development of ALS [2]. Evidence from animal studies and clinical trials has shown an increase in the numbers of peripheral monocytes and central nervous system microglia, with a high level of inflammatory cytokines in ALS; these phenomena of inflammation have neuroprotective effects against ALS during the early phase but become neurotoxic in advanced stages [2, 3]. Additionally, a positive correlation between several ALS-associated mutations and enhanced inflammatory responses was observed in ALS patients and transgenic mice, indicating that dysfunction of the immune system might play a role in the pathogenesis of ALS [4–6].

Although there is controversy about the autoimmune theory in ALS [7, 8], cases of ALS patients with a diagnosis of autoimmune disease (AID) have been widely reported [9–16], suggesting a possible association between ALS and AIDs. The significantly higher incidence of AIDs in ALS patients demonstrated by several large population studies also provides evidence to support this hypothesis [17–19]. However, it is far from clear whether this is a simple coexistence or causal relationship between ALS and AIDs, and more research is needed to obtain a deeper understanding. To date, there is little research about the clinical characteristics and serological inflammatory biomarkers of ALS patients with AIDs, and it remains unclear whether there are differences between the prognosis of patients with AIDs and those without AIDs. On the other hand, there is currently no effective treatment for ALS, and immunotherapy has been used in ALS patients with AIDs according to the theory that autoimmunity is involved in the pathogenesis of ALS, although there is little evidence of immunotherapy being effective in ALS [9–11, 14]. The underlying mechanism is still unknown, and a possible explanation is the severity of inflammation in AIDs. Immunotherapeutic strategies have mostly been applied to ALS patients with connective tissue diseases such as rheumatoid arthritis (RA) [11–13], systemic lupus erythematosus (SLE) [9, 10], and Behcet’s disease (BD) [14, 15] in previous studies, and these studies have resulted in conflicting findings; thus, the autoimmune mechanism in these ALS patients with concomitant AIDs needs further exploration.

Thus, this study investigated the incidence of AIDs in Chinese ALS patients and compared the clinical features and serum inflammatory factor levels between patients with and without AIDs to explore the relationship between ALS and AIDs. Furthermore, the survival in ALS patients with concomitant AIDs was also compared with those without concomitant AIDs, and the association between the history of immunotherapy and prognosis in patients with AIDs was also studied to better understand the mechanism of coexisting ALS and AIDs.

## Methods

We performed a retrospective study based on the ALS Registry dataset, which includes a cohort of 1655 ALS patients registered at Peking Union Medical College Hospital from October 2013 to December 2020. Patients who fulfilled the revised El Escorial criteria for clinically definite, probable or laboratory-supported probable ALS were included in the dataset. Age at onset, sex, disease duration (time from onset to registration), predominant involvement of upper or lower motor neurons (UMN or LMN, respectively), sites of onset, revised ALS functional rating scale (ALSFRS-R), family history, and other medical history were collected at the time of registration. The rate of disease progression (DPR) was calculated with the following formula: (48—ALSFRS-R)/time from disease onset to registration. We defined rapid disease progression as DPR ≥1 according to previous studies, and slow disease progression was defined as DPR<1 [20, 21]. Data on laboratory indexes were obtained by screening hospital statistics, most of which were obtained from inpatient records during hospitalization. All the patients were followed up through telephone or clinic visits at least once every year to assess and re-evaluate their clinical symptoms and ALSFRS-R. The primary endpoints, including death and tracheostomy, were recorded during follow-ups. The study was approved by the Research Ethics Committee of Peking Union Medical College Hospital, and consent was provided by all the participants.

### Participants

We reviewed all the medical records of autoimmune disorders in patients from our dataset. The clinical features and inflammatory biomarkers of patients with and without autoimmune disorders were compared. To ensure the accuracy of the diagnosis that excluded autoimmune diseases in the control group and considering the integrity of serological data, we consecutively included hospitalized patients as the control group. Considering the heterogeneity of clinical manifestations and prognosis of patients with gene mutations (such as slow progression in patients with SOD1 H46R and relatively rapid disease course in patients with FUS P525L), we excluded patients with familial ALS. Patients with acute infection, liver and kidney diseases, immunosuppression, and malignant tumors were also excluded. Serum inflammatory parameters, including neutrophil count (NC), lymphocyte count (LC), neutrophil-lymphocyte ratio (NLR), hypersensitive C-reactive protein (hs-CRP), and erythrocyte sedimentation rate (ESR), were collected. We also collected records of immunotherapy, including treatment with immunosuppressants, steroids, and intravenous immunoglobulin, during the course of ALS. Patients were followed up until December 2020, and overall survival was calculated as the time from disease onset to endpoints or last follow-up.

### Statistical analysis

All analyses were performed with SPSS Version 22.0 software, and figures were generated using GraphPad Prism Version 7.0 software. Patients were grouped according to whether they were diagnosed with AIDs. Considering the small proportion of patients with AIDs in our cohort, we used Propensity-score matching to balance the covariates, and 1:1 matching was conducted between the patient group (ALS patients with concomitant AIDs) and the control group (ALS patients without a history of AIDs). A logistic regression model was performed in which the covariates were sex, onset age, and disease duration. Clinical parameters and serum biomarkers were compared before and after matching the patients. Student’s t test or Mann–Whitney U test was used for continuous variables, while Fisher’s test or chi-square test was used for categorical variables. Survival analyses were performed with the Kaplan–Meier method and log-rank test to investigate the prognosis of ALS patients with AIDs compared with that of controls. Moreover, survival was compared for all patients categorized according to clinical features, including sex, age of onset, sites of onset, rate of disease progression, and predominant involvement of UMN or LMN. A Cox regression hazard model was also used to estimate the hazard ratio of immunotherapy in the survival of ALS patients after adjusting for relevant factors. A *p value* of < 0.05 was considered significant.

## Results

We found 26 (1.6%) ALS patients with AIDs in our dataset. Table 1 shows the details of these patients with each autoimmune disorder. The number of patients with Sjögren syndrome (SS) was highest in this ALS cohort, followed by the number of patients with asthma and Graves’s disease. The diagnosis of AIDs was mostly made before the onset of ALS symptoms (65.4%), except for several cases of SS, Hashimoto, and primary biliary cirrhosis that were diagnosed after the onset of ALS. The mean age at onset was 55.27±7.94 years old in these patients. Bulbar onset was found in 4 (15.4%) patients, while 22 (84.6%) presented with spinal onset. There were some noteworthy and uncommon clinical features in this cohort, including ataxia (7.7%), tremor (3.8%), subjective sensory complaints (3.8%), and facial and extraocular muscle weakness (7.7%). Among these patients with AIDs, 8 (26.9%) were treated with immunotherapy, and half of them were diagnosed with SS. Of all patients who received immunotherapy, corticosteroids and intravenous immunoglobulin were most commonly used, followed by cyclophosphamide.

**Table 1:** Summary of autoimmune diseases in patients with ALS.

Autoimmune disease	Patients with ALS, n (%)	Patients with immunotherapy, n
Asthma	5 (0.302)	1
Graves’s disease	3 (0.181)	0
Hashimoto	2 (0.121)	0
Rheumatoid arthritis	2 (0.121)	1
Sjögren syndrome	6 (0.363)	4
Primary biliary cirrhosis	1 (0.060)	1
Ulcerative colitis	1 (0.060)	0
Scleroderma	1 (0.060)	0
Myasthenia gravis	1 (0.060)	1
Vitiligo	2 (0.121)	0
Chronic urticaria	2 (0.121)	0
Total	26 (1.571)	8

### Comparison of clinical features and serum inflammatory factors between ALS patients with or without a history of AIDs

Out of the 110 ALS inpatients (63 males, 47 females) who denied a history of AIDs, 26 were selected to match those with sex, onset age, and disease duration. The clinical characteristics and serum inflammatory parameters were compared between groups and are presented in Table 2. Before propensity- score matching, the median age at onset was older in ALS patients with AIDs than in those without AIDs (*p*<0.01). The median DPR was higher in patients with autoimmune disorders, but the results did not reach statistical significance. However, no significant difference was found in terms of sex, sites of onset, disease duration, ALSFRS-R score, or involvement of UMN or LMN between patients with and without AIDs, except for a significantly lower ALSFRS-R respiratory score in patients with AIDs. Although the levels of hs-CRP, ESR, and NLR were higher in patients with AIDs, the results were not obviously different between the two groups. After propensity-score matching by sex, onset age, and disease duration, ALS patients with a history of AIDs and those without a history of AIDs had similar onset ages and sex ratios. There were also no significant differences between the two groups in terms of disease duration, ALSFRS-R score, predominant involvement of UMN or LMN, and serum inflammatory factors. The ALSFRS-R respiratory score remained significantly different between the two groups (*p*<0.05).

**Table 2:** Clinical characteristics and serum inflammatory biomarkers of ALS patients and controls.

	Before matching			After matching		
	SALS patients with autoimmune diseases (n = 26)	Control SALS patients (n = 110)	*P*	SALS patients with autoimmune diseases (n = 26)	Control SALS patients (n = 26)	*P*
Age of onset (years)^a^	55.27±7.94	49.08±12.10	**0.002**	55.27±7.94	52.31±12.16	0.304
Sex			0.502			0.402
Males	13(50.0%)	63(57.3%)		13(50.0%)	16(61.5%)	
Females	13(50.0%)	47(42.7%)		13(50.0%)	10(38.5%)	
Disease duration (months) ^b^	14.0(8.0, 19.5)	11.0(6.8, 18.3)	0.320	14(8, 19.5)	9(6.8, 18.3)	0.256
Sites of onset			0.466			0.308
Bulbar	4(15.4%)	24(21.8%)		4(15.4%)	7(26.9%)	
Spinal	22(84.6%)	86(78.2%)		22(84.6%)	19(73.1%)	
Predomint involvement of UMN or LMN			0.101			0.087
UMN	7(26.9%)	49(44.5%)		7(26.9%)	13(50.0%)	
LMN	19(73.1%)	61(55.5%)		19(73.1%)	13(50%)	
ALSFRS-R total score ^b^	39(35, 43)	39(36, 43)	0.626	39(35, 43)	39(35, 43)	1
Bulbar score of ALSFRS-R ^b^	12(11, 12)	12(10, 12)	0.299	12(11, 12)	12(10, 12)	0.463
Respiratory score of ALSFRS-R ^b^	12(10, 12)	12(12, 12)	0.001	12(10, 12)	12(12, 12)	0.032
DPR ^b^	0.64(0.33, 1.2)	0.63(0.38, 1.12)	0.605	0.64(0.33, 1.2)	0.93(0.31, 1.58)	0.51
NC (×109/L)^b^	3.32(2.65, 3.73)	3.35(2.74, 4.19)	0.341	3.32(2.65, 3.73)	3.04(2.47, 4.39)	0.865
LC (×109/L)^b^	1.66(1.23, 2.21)	1.72(1.46, 2.07)	0.58	1.66(1.23, 2.21)	1.62(1.46, 2.14)	0.992
NLR^b^	1.98(1.5, 2.7)	1.83(1.41, 2.50)	0.884	1.98(1.5, 2.7)	1.74(1.39, 2.30)	0.696
hs-CRP (mg/L)^b^	1.16(0.66, 1.7)	1.06(0.43, 1.75)	0.924	1.16(0.66, 1.7)	0.88(0.40, 1.70)	0.443
ESR (mm/h)^b^	8.0(3.5, 14.0)	6(3.0, 9.0)	0.16	8.0(3.5, 14.0)	7.5(4.0, 8.8)	0.429

^a^ mean ± SD.

^b^ median (interquartile range).

UMN, upper motor neuron; LMN, lower motor neuron; ALSFRS-R, revised ALS functional rating scale; DPR, disease progression rate; NC, neutrophi counts; LC, lymphocytes counts; NLR, neutrophil-lymphocyte ratio; hs-CRP, hypersensitive C-reactive protein; ESR, erythrocyte sedimentation rate

### Overall survival of ALS patients

**Fig 1:**
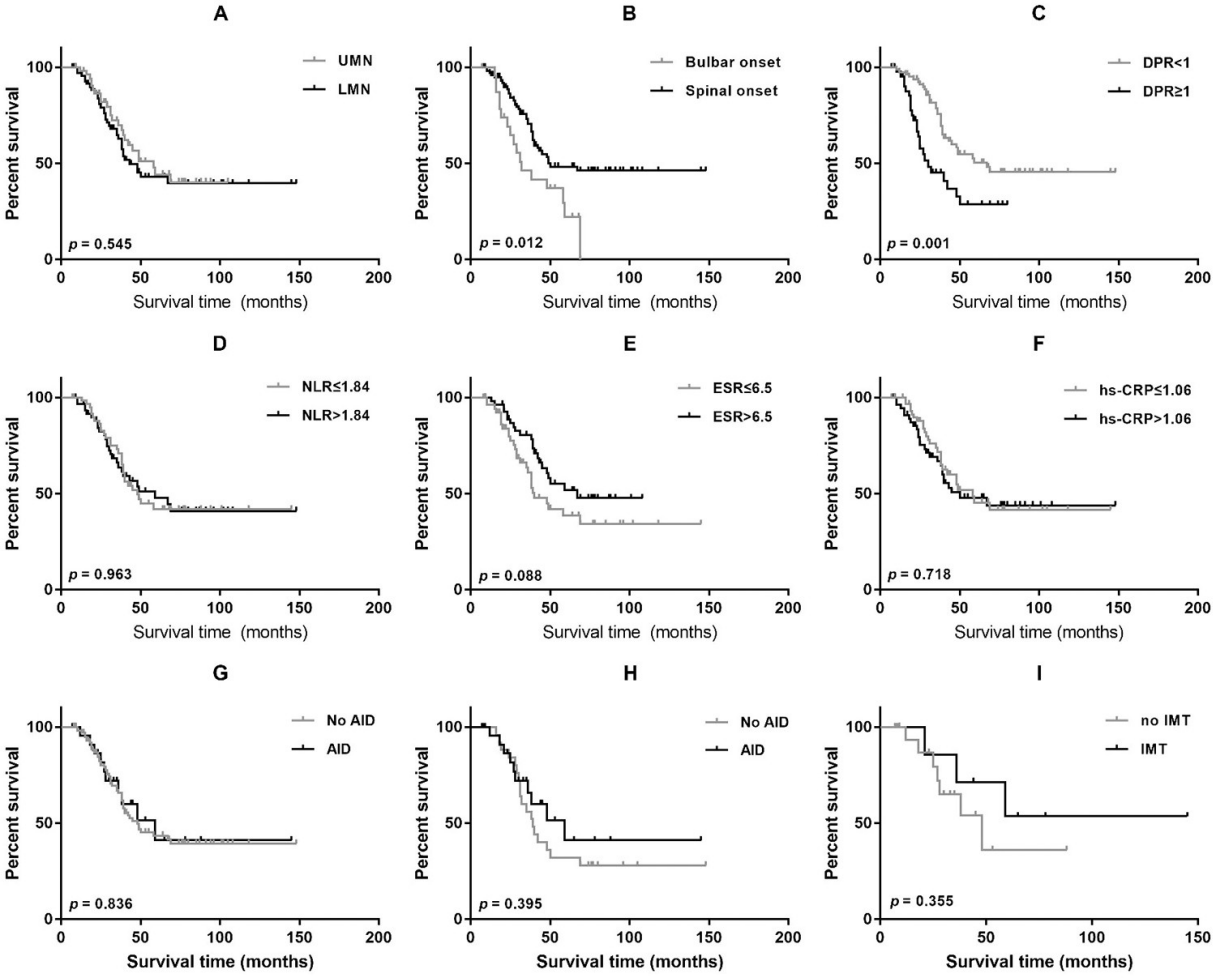
The effect of clinical parameters and immunotherapy on survival. Survival curves of ALS patients categorized by predominant involvement of UMN or LMN (A), site of onset (B), DPR (C), NLR (D), ESR (E), hs-CRP (F), coexisting AID (G), and immunotherapy (H-I). Patients with bulbar onset and rapid disease progression had worse survival when the relationship between clinical features and survival was analyzed (A-C). No significant difference in survival was found in patients grouped by serum inflammatory biomarkers. Coexisting autoimmune diseases do not influence the survival in ALS patients before and after propensity score matching (G-H). The patients with coexisting ALS and AIDs who had a history of immunotherapy presented a trend of longer survival. UMN, upper motor neuron; LMN, lower motor neuron; DPR, disease progression rate; NLR, neutrophil-lymphocyte ratio; ESR, erythrocyte sedimentation rate; hs-CRP, hypersensitive C-reactive protein; AID, autoimmune disease; IMT, immunotherapy.

Of 136 ALS patients enrolled, 9 were lost to follow-up, and 59 (43.4%) reached the endpoint. The mean survival time from disease onset was 45.0 months (95% CI 33.0–65.0 months). Kaplan–Meier analysis showed that patients with bulbar onset had shorter overall survival than those with spinal onset (log-rank test *p* = 0.012; Fig 1B). Patients with rapid disease progression also had a shorter survival time than those with a lower rate of disease progression (log-rank test *p* = 0.001; Fig 1C). No difference in terms of overall survival time was found when patients were grouped based on the predominant involvement of UMN and LMN (Fig 1A). Using the median as a cutoff point, we observed no significant difference in overall survival time among patients categorized according to the levels of inflammatory biomarkers, but patients with a higher level of ESR at baseline had a longer survival time (hs-CRP, ESR, and NLR; Fig 1D–1F).

The correlation between coexisting AIDs and overall survival was also analyzed. The median survival from the time of disease onset was 59.0 months (95% CI: 29.4–88.6 months) in the group of ALS patients with coexisting AID and 48.0 months (95% CI: 31.3–64.7 months) in the control group (log-rank test *p* = 0.836; Fig 1G). There was still no significant difference in survival between ALS patients with and without a history of AIDs after propensity- score matching (log-rank test *p* = 0.395, Fig 1H). Multivariate analysis showed that older age of onset, faster disease progression, and decreased level of ESR were associated with shorter survival (Table 3).

**Table 3:** Multiple regression analysis of prognostic factors for overall survival in the whole ALS patients.

Variable	B	Exp(B)	95%CI	*P*
Age at onset	0.029	1.03	1.002–1.058	0.034
Site of onset	-0.507	0.603	0.300–1.210	0.155
DPR	1.267	3.550	1.817–6.936	<0.001
hs-CRP	0.44	1.552	0.833–2.893	0.167
ESR	-0.876	0.416	0.224–0.775	0.006
NLR	0.035	1.036	0.565–1.899	0.910
History of autoimmune disease	-0.324	0.723	0.293–1.783	0.481

DPR, disease progression rate; hs-CRP, hypersensitive C-reactive protein; ESR, erythrocyte sedimentation rate; NLR, neutrophil-lymphocyte ratio.

### Subgroup analysis on the prognosis of SALS patients with coexisting AIDs

**Fig 2:**
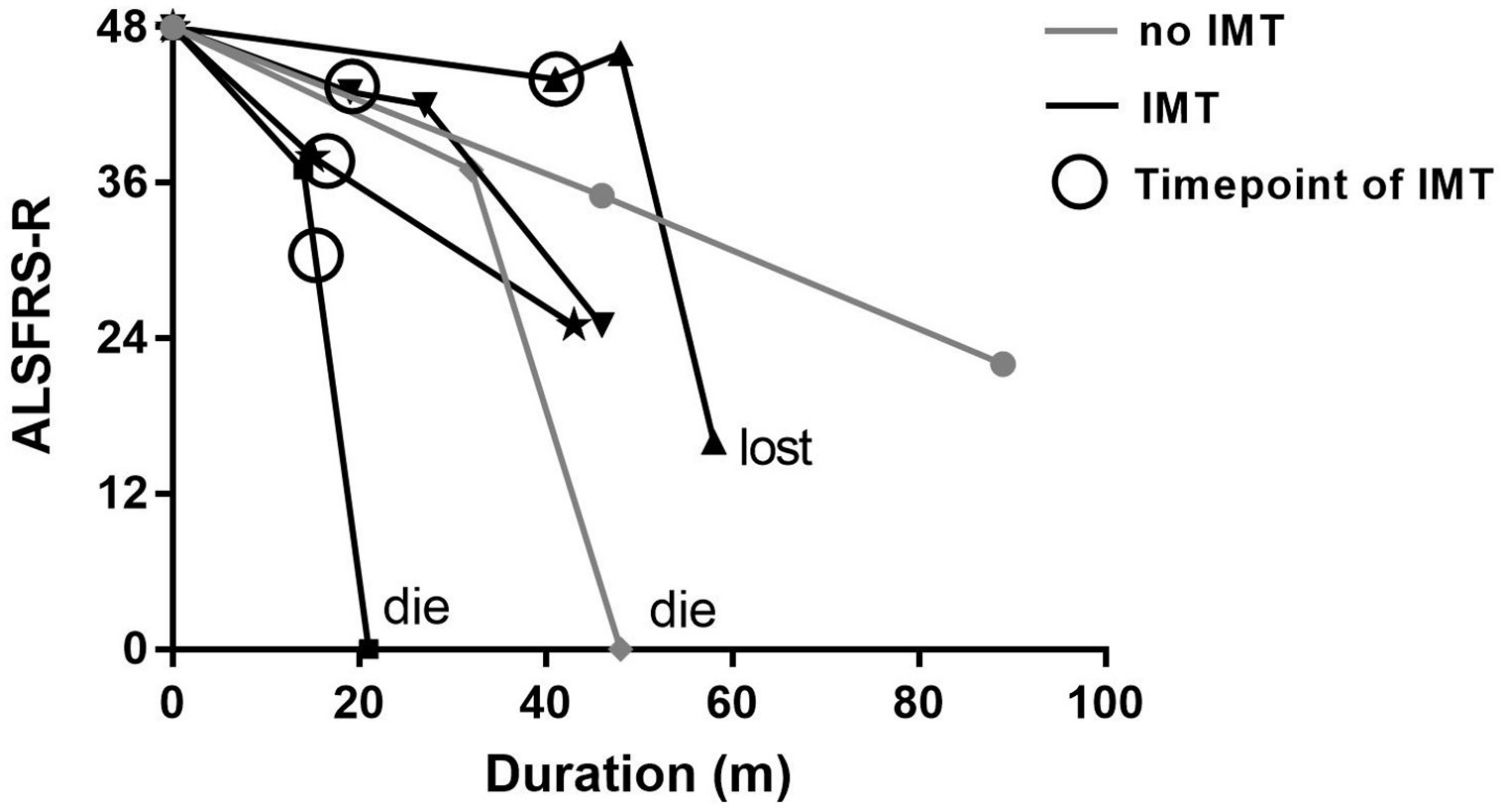
Variation in the ALSFRS-R score among ALS patients with coexisting Sjögren syndrome. In patients who received immunotherapy (blank line), the ALSFRS-R score increased in 1 patient and declined at a slower rate after treatment in 2 patients. The slope of decreased ALSFRS-R score increased in patients not treated with immunotherapy (gray line). ALSFRS-R, revised ALS functional rating scale; IMT, immunotherapy.

Eight SALS patients with coexisting AIDs were treated with immunotherapy, and half of them suffered from SS. We analyzed the changes in the ALSFRS-R scores in patients with SS according to the time point of treatment. Among four SALS patients treated with immunotherapy, improvement in the ALSFRS-R score was observed for one patient (44 vs. 46), two had a lower rate of disease progression after treatment (0.263 vs. 0.125; 0.667 vs. 0.464), and one died four months after treatment (Fig 2). However, it should be noted that in patients treated with immunotherapy, the ALSFRS-R scores further decreased rapidly after several months. Moreover, variation in the ALSFRS-R score was also observed in the other two SALS patients who had not been treated with immunotherapy, and the ALSFRS-R scores decreased gradually during follow-ups (0.283 vs. 0.302 for one patient; another patient died 16 months after registration; Fig 2).

Ten patients with AIDs reached the endpoints, three of whom had a history of immunotherapy. There were no significant differences between the two groups in terms of onset age, sex, sites of onset, predominant involvement of UMN or LMN, ALSFRS-R score, DPR and serum inflammatory factors (Table 4). The disease duration was significantly longer in SALS patients who had a history of immunotherapy (*p*<0.05). Kaplan–Meier analysis showed longer survival of ALS patients with AIDs who were treated with immunotherapy (96.36±21.03 months) compared with those who were not treated with immunotherapy (52.34±9.11 months), but the difference between groups was not significant (Fig 1I).

**Table 4:** Clinical characteristics and serum inflammatory biomarkers of ALS patients with AID grouped by the history of immunotherapy.

	ALS plus AID patients treated without immunotherapy (n = 18)	ALS plus AID patients treated with immunotherapy (n = 8)	P value
Age of onset (years)	54.00±8.52	58.13±5.96	0.229
Sex (Males)	10(55.6%)	3(37.5%)	0.394
Sites of onset (Bulbar)	3(16.7%)	1(12.5%)	0.782
Predomint involvement of LMN	14(77.8%)	5(62.5%)	0.425
Disease duration (months)	9.50(7.50, 15.00)	18.50(14.25, 36.00)	0.034
ALSFRS-R	37.89±7.87	37.88±4.64	0.996
ALSFRS-R-bulbar score	10.44±2.91	11.63±0.74	0.275
ALSFRS-R-respiratory score	10.89±1.49	10.88±1.81	0.984
DPR	0.66(0.39, 1.47)	0.46(0.19, 0.76)	0.232
NLR	2.07(1.50, 2.70)	1.85(1.41–3.09)	0.923
hs-CRP	1.16(0.61, 1.70)	1.13(0.84, 2.62)	0.698
ESR	7.00(2.75, 14.00)	10.00(7.00, 21.00)	0.313

LMN, lower motor neuron; ALSFRS-R, revised ALS functional rating scale; DPR, disease progression rate; NLR, neutrophil-lymphocyte ratio; hs-CRP, hypersensitive C-reactive protein; ESR, erythrocyte sedimentation rate

## Discussion

Our study evaluated the epidemiology and phenotype of ALS patients with AIDs in China and found a higher prevalence of autoimmune disease (myasthenia gravis, primary biliary cirrhosis, scleroderma, SS, and ulcerative colitis) in ALS patients than in the general population [22–26], which is similar to the results of previous epidemiological studies [17, 18]. A study of exclusively England-registered hospital records showed a higher rate of ALS than expected in patients with a prior diagnosis of AIDs, such as asthma, celiac disease, myasthenia gravis, SS, SLE, and ulcerative colitis [17]. Another nationwide case–control study in Sweden found that patients with ALS had a 47% higher risk of previously being diagnosed with AIDs, including myasthenia gravis, dermatomyositis, multiple sclerosis, hypothyreosis, and Guillain–Barre syndrome [18]. These findings supported the potential association between ALS and AIDs, while the underlying mechanism had not been determined.

The cooccurrence of AIDs and ALS is mostly explained by probable shared pathological mechanisms and genetic susceptibility [27, 28]. Dysfunctional immune regulation and increased inflammatory responses in both AIDs and ALS, including dysfunctional lymphocytes, increased proinflammatory factor production, and complement activation, are commonly suggested to explain the coexistence of ALS and AIDs [29–31]. Our study also observed a slightly higher level of serum inflammatory biomarkers in ALS patients with coexisting AIDs. Additionally, the higher risk of AIDs in individuals with parents who are diagnosed with ALS suggests the probable contribution of genetic factors [32, 33]. However, other studies found inconsistent results and failed to observe an increased risk of AIDs in children of ALS patients [18], indicating the involvement of other factors. Although some studies detected autoantibodies in patients with ALS [34, 35], ALS patients fail to respond to immunotherapy clinically [11, 14], and ALS is not an autoimmune disease. However, some previous cases with coexisting ALS and AIDs have been reported to respond to immunotherapy [9, 36], whose symptoms were reversed or stabilized after immunotherapy. Although none of the ALS patients with a history of AIDs in this study continued to improve or stabilize after immunotherapy, we found that the survival time was slightly longer in those with a history of immunotherapy. These findings confirm the potential role of autoimmunity in the mechanism of ALS patients with coexisting autoimmune diseases, and autoimmune diseases and ALS may not simply occur concomitantly by chance. Thus, we assume that autoimmune diseases may be involved in the pathogenesis of ALS, but the specific pathological mechanism is still unclear. Moreover, the differences in the efficacy of immunotherapy in patients with coexisting ALS and autoimmune diseases [12, 14, 15, 37] indicate the complexity of immune dysfunction in these specific patients, which requires further study in the future.

Most current studies on the clinical characteristics of ALS with AIDs have been presented in the form of case reports, while our study provides a valuable summation and comparative analysis of this rare and special cohort of ALS patients. Our study found that SALS patients with AIDs had an older age at onset than those with pure ALS. Although it is commonly accepted that autoimmune diseases are more prevalent in young women, epidemiologic studies have found that different autoimmune diseases have different predilection ages of onset. Older adults tend to suffer from Sjogren disease, rheumatoid arthritis, dermatomyositis, and primary systemic vasculitis [38], which are more prevalent in ALS patients, as shown in previous studies and this study. Another possible explanation of the older onset age in ALS patients with coexisting AIDs is aging-related immune dysregulation [39, 40]. Assuming the contribution of autoimmune diseases to the occurrence of ALS, increased inflammatory and oxidative stress with aging may lead to susceptibility to ALS in elderly individuals. Moreover, we found more severe respiratory function in ALS patients with a history of AIDs than in those without. The difference remained significant after matching for onset age, sex, and disease duration, indicating early involvement of the respiratory system in these patients with coexisting ALS and AIDs. Pulmonary involvement is a common feature in autoimmune diseases, mostly presenting with airway disease, dysfunction of respiratory muscle, and interstitial lung disease. Respiratory disturbances in ALS patients with coexisting AIDs might partly be attributed to autoimmune diseases. Moreover, severe inflammation in ALS patients with coexisting AIDs may cause early involvement of respiratory function. Pronto-Laborinho A et al. [41] found a correlation between increased IL-6 levels and a reduction in phrenic nerve CMAP amplitude, indicating the possible role of inflammatory factors in respiratory dysfunction in ALS. Unfortunately, we do not have relevant information about these patients’ respiratory function to verify these speculations, which may need further investigation.

Another interesting finding in this study is the positive correlation between ESR and survival in ALS patients. Previous studies [42, 43] suggested an association between elevated levels of systemic inflammatory factors and poor prognosis in ALS. Such contradictory findings may be explained by the different durations of ALS in previous studies and this study. Inflammation in the early stage might play a protective role in the pathophysiology of ALS, while excessive inflammation during the advanced stage contributes to disease progression and poor prognosis [44, 45]. The patients with ALS included in this study might be in the early stages of the disease, in which a high level of ESR would be a positive indicator of better prognosis. Another alternative interpretation is the interference of immunotherapy, which may lead to an increased ESR in ALS patients. The trend of longer survival in patients with a history of immunotherapy could result in a better prognosis in patients with a higher level of ESR. Additional speculation is the possible particular pathways or mechanism in neuroinflammation that the ESR may be involved, which may need further exploration. The exact prognostic role of ESR in ALS patients warrants more research with a larger sample size.

Although patients with ALS had a higher prevalence of AIDs than the general population, no significant difference in survival was found in our study when patients with AIDs were compared with patients without AIDs. These results may indicate that the inflammatory process in AIDs does not promote disease progression or shorten the life expectancy of ALS patients. We hypothesized that AIDs probably play an essential role in the pathogenesis of ALS; nevertheless, they have little influence on prognosis. Further investigations are needed to confirm and explain the observations.

There were still several limitations in our study. First, this study was performed by retrospective investigation, leading to limitations in terms of the information we could retrieve, especially data about AIDs and changes in clinical symptoms related to treatments. The rate of AIDs might be underestimated in patients with ALS. Second, different types of AIDs were studied as a whole due to the relatively low prevalence of AIDs in patients with ALS, which limited the reliability and validity of the results from the study of the association between AIDs and survival of ALS patients. Similarly, the number of patients treated with immunotherapy was small, and more patients should be studied to confirm the effect of immunotherapy on ALS patients with AIDs. Third, the patients included in our study were recruited from a single third-level medical center in China, and we could not estimate the actual prevalence of Chinese ALS patients with AIDs. Moreover, the shortage of representatives also restricts generalization and application of our results to other ethnic groups. Thus, more studies of different populations are needed to verify and confirm the association between ALS and AIDs.

## Conclusion

In conclusion, this study investigated the clinical features of SALS patients with coexisting autoimmune diseases and showed that these patients had an older age of onset and earlier involvement of respiratory function than patients without AIDs. Moreover, the combination of autoimmune diseases did not affect the survival of patients with ALS, while some patients with a history of immunotherapy had a trend of longer survival. The therapeutic effect of immunotherapy in ALS patients with coexisting autoimmune diseases deserves further follow-up and observation. This study was the first to summarize the clinical characteristics and prognosis of ALS patients with coexisting autoimmune diseases, which may shed some light on the relationship between ALS and autoimmune disorders. Clinicians should enhance their awareness of the early diagnosis of AIDs in ALS patients and provide comprehensive treatments for AIDs to improve the prognosis of these patients.
